# Investigating the correlation between organizational cynicism and teacher burnout: an evidence from TRNC secondary public school teachers

**DOI:** 10.3389/fpsyg.2025.1674083

**Published:** 2025-11-26

**Authors:** Bahar Karaman, Zehra Altınay, Fahriye Altinay, Gokmen Dagli, Rustam Shadiev, İslam Suiçmez

**Affiliations:** 1 Faculty of Education, Yakin Dogu Universitesi, Nicosia, Cyprus; 2 College of Education, Girne Universitesi, Girne, Cyprus; 3 Faculty of Education, Zhejiang University, Hangzhou, China

**Keywords:** trnC, secondary school teachers, organizational cynicism, burnout, correlation, regression

## Abstract

This study aimed to explore the correlations between organizational cynicism, burnout, and socio-demographic variables. The sample consisted of 553 Turkish Republic of Northern Cyprus (TRNC) secondary public-school teachers. The Maslach Burnout Inventory, and the Organizational Cynicism Scale, were employed to obtain data from the participants. Results demonstrated that the cognitive and reduced personal accomplishment dimensions had the highest mean scores. Furthermore, all socio-demographic variables were correlated with burnout among the participating teachers. Conversely, organizational cynicism revealed statistically significant differences and correlations with gender, education level, and seniority variables. Spearman’s rank correlation revealed a significant and positive relationship between the two constructs, with a small-to-moderate effect size. Additionally, a simple linear test indicated that organizational cynicism had a significant and positive impact on burnout, explaining 9.9% of the total variance. Although the predictive power is limited, the findings suggest that interventions targeting cynical attitudes in school environments may help to mitigate burnout among teachers. Also organizational cynicism was also linked to seniority and educational level, indicating that tailored professional support and school-based climate interventions may be necessary for different teacher groups to prevent the escalation of burnout.

## 1 Introduction

Several authors have signified that teaching is a stressful profession (Allahkerim, 2020; Mousavy and Nimechsalem, 2014). Shirazizadeh and Moradkhani (2018) posit that teachers play a prominent role in determining the skills and abilities of the learners, and thus direct them to related fields that fits their talents. Therefore, teachers shape the fate of the young generation, which in turn triggers their level of stress. In addition, numerous researchers have indicated that teachers are more likely to experience burnout syndrome compared with other professions (Gülşen, 2025; Jones et al., 2003; Kinman et al., 2011). Studies have explored the main factors that cause burnout among teachers. Although the findings are scattered, researchers have reached a consensus regarding the most vital factor that stimulates burnout syndrome, simply expressed as a “chronic stress.” The other factors that elevate burnout include feeling excessive pressure while teaching learners, over-crowded learning spheres, low student motivation, and disrespectful behaviors by learners (Kinman et al., 2011). Furthermore, Atmaca (2016) reported excessive workload, difficulty dealing with intensive responsibilities, such as forming a curriculum and satisfying the demands of educational stakeholders, and establishing intensive effort to meet educational goals as other influential factors.

Similar to in many organizations, organizational cynicism is a crucial notion in educational organizations. Teachers with positive feelings and attitudes towards to their education institutions may build strong emotional ties, work in a further productive manner, be more likely to accept the values, and take intensive efforts to reach educational goals (Akar, 2018). Organizational cynicism reflects teachers’ negative attitudes toward administrators, students, and colleagues, which adversely affect organizational performance. Since educators play a key role in the teaching–learning process, teachers are central to students’ academic success (Allahkerim, 2020). Mukundan and Khandehroo (2011) stressed that when teachers experience burnout, they are more likely to have negative impact on students’ academic performance, which reduces the quality of teaching. Furthermore, organizational cynicism is also considered the main reason for low teacher motivation, reduced dedication, and low teacher engagement. Furthermore, these diminish school effectiveness and block the education institution from achieving its goals. School principals and educational administrators should better understand teacher burnout and organizational cynicism and take appropriate measures to address their causes within schools.

Therefore, this study had two aims: to examine the relationships between the two concepts and evaluate the levels of burnout and organizational cynicism among participating teachers regarding significant differences consistent with certain socio-demographic variables (i.e., gender, age, marital status, education level, and seniority).

Educational institutions are considered as crucial institutions for the progress and development of societies. Hence, teachers in these institutions constitute the most important potion of human resources. Therefore, teachers should be happy and motivated to teach for the future of society, as unhappy and demotivated teachers cannot teach effectively (Vatansever-Bayraktar and Mutlu, 2023). Such teachers may prevent students to achieving an adequate level of education, which may lead failure in recognizing their potential among future generations. Teachers may feel unhappy due to various reasons. Teacher burnout and organizational cynicism are important factors that may lead teachers to feel unhappy, demotivated, and experience poor sense of belonging. Therefore, conducting research to determine the levels of teacher burnout and organizational cynicism is necessary to develop necessary strategies to generate welcoming and peaceful school climate to minimize the influencing factors as well as maximize teachers’ effectiveness for generating necessary grounds for qualified education. Hence, this study is significant as the findings aim to inform education administrators and advance their understanding of factors that cause burnout and organizational cynicism. Furthermore, the results may assist them in understanding the association between teacher’s burnout and organizational cynicism. In addition, a literature review revealed that limited studies examined the correlation between these variables. Thus, this study contributes to literature through its findings and recommendations to educational administrators as well as future researchers to create better understanding regarding the notions in education context.

Despite the extensive research on the connection between burnout and organizational cynicism across various educational settings, there remains a notable absence of empirical studies from regions with unique sociocultural and administrative frameworks, such as the Turkish Republic of Northern Cyprus (TRNC). The TRNC offers a distinct institutional environment characterized by specific teacher workloads, policy execution, and organizational climate, setting it apart from many Western and larger national education systems. By situating this study within the broader international discourse on burnout and cynicism, it not only fills a geographical void but also enhances cross-cultural insights by illustrating how these phenomena are expressed within a smaller, semi-autonomous educational context.

### 1.1 Organizational cynicism

Cynicism refers a set of negative beliefs and attitudes of the employees towards to their organizations, which results thoughts of the existence of dishonesty within the organizational sphere. Cynicism emerged from the Greek work “kyon” that stands for dog or the town of Cynosarges, where the cynics supposedly had schools (Brandes et al., 1998). Many scholars have focused on the main reasons that cause organizational cynicism. Sağır and Oğuz (2012) reported burnout syndrome, low job satisfaction, poor leadership, and organizational injustice as main reasons that generated organizational cynicism.

Brandes et al. (1998) are considered as pioneers of the term organizational cynicism. According to them, organizational cynicism has three sub dimensions: cognitive, affective, and behavioral. The cognitive dimension concentrates on the employee perceptions regarding the existence of dishonesty within the organization, whereas affective organization focuses on employees’ unpleasant feelings towards to their colleagues. The behavioral dimension acts as an outcome of the cognitive and affective dimensions (Güllü and Çoruk, 2022). Nous (2007) stressed that managers should take necessary measures towards to organizational cynicism, as chronic cynicism may result in excessive financial burden, which may endanger the organization’s sustainability. Gök and Ünal (2021) highlighted poor organizational level, decreased productivity, and job dissatisfaction as outcomes of organizational cynicism.

Various researchers have attempted to explore the outcomes of organizational cynicism in education institutions. Yazıcıoğlu and Gençer (2017) indicated that destructive criticism, less efforts to reach educational goals, and humiliation were the most common outcomes of organizational cynicism in the education sector. When faced with organizational cynicism, teachers may stop offering suggestions, believing their opinions to improve education are ignored. Furthermore, they may feel no equity and justice in the school, and may lose hope regarding the future of their school (Kalağan and Aksu, 2010). Vatansever-Bayraktar and Mutlu (2023) reported that organizational cynicism in education institutions lowered teachers’ motivation to teach, which diminished their performance and lowered the quality of education and students’ academic performance.

### 1.2 Burnout

Burnout can be defined as “a loss of energy day by day.” However, when viewed as a term in professional life, burnout is the main driver that hampers productivity and negatively impacts the capacity of an organization (Hurşitoğlu, 2017).

A literature review on burnout revealed that the notion was first discussed in 1961 by Greene in a novel titled “A Case of Burnout.” This novel narrates that an architect had a mental breakdown and escaped to the forests in the African continent. In this novel, the concept of burnout was explained as “the individual, feeling intensely weak and exhausted, being full of grudge and anger towards to the architectural profession and then losing his passion towards to being architect” (Koç, 2019). Other than this notion, burnout was first explained by Herbert Freudenberger, a psychologist, in 1974. Freudenberger published an article called “Staff Burn-out” and described the burnout as “becoming exhausted by making excessive demands on energy, strength or resources within the workplace” (Freudenberger, 1974). Polman et al. (2010) indicated that long lasting stress may lead burnout. Burnout was an outcome of negative emotional state caused by exhausted employees aiming to prove themselves to their colleagues and superiors (Edú-Valsania et al., 2022). Numerous scholars have proposed different models to deepen understanding towards burnout. Maslach, a reputable psychologist, conducted several studies with her colleagues during 1970–1980 (Heinemann and Heinemann, 2017). She argued that burnout syndrome comprised three crucial dimensions: emotional exhaustion, depersonalization and reduced personal accomplishment (Bakker et al., 2014). Emotional exhaustion represents feelings and sensation of being exhausted of the psychological efforts taken and lack of emotional energy for work related tasks, which increases tiredness and fatigue. Depersonalization refers to the interpersonal component of burnout and denotes the negative attitudes and behaviors, loss of idealism as well as isolation from building interpersonal relationship with other people. Reduced personal achievement focuses on destructive professional self-evaluations and having doubts regarding one’s capabilities necessary to work in a productive manner. Low morale and decrease in productivity level are signs of reduced personal achievement (Edú-Valsania et al., 2022).

Subon and Sigie (2016) indicated the signs of teacher burnout and highlighted that these teachers tended to:

- feel more tired.
- experience a chronic headache.
- experience muscle aches.
- have sleep disorders.
- experience terrible back pain.
- lose their motivation and passion towards to the teaching profession.
- encounter problems with their students and colleagues.
- omit their responsibilities.
- to leave early.

Several authors have attempted to explore the main factors that impact teacher motivation. Allahkerim (2020) reported that experiences of role ambiguity, challenges in educational facilities, feelings of isolation, inadequate support by school principals, lack of training and development opportunities, safety problems at school, and centralized management style in education institution were the main sources of teacher burnout. Scott (2019) indicated that emotional problems, poor interaction and communication with colleagues and students, experiences of problems in school, and negative attitudes towards to their professional career were some key factors that caused burnout. Others scholars indicated that poor salary, lack of ventilation and lighting, being forced to purchase teaching materials by the school principals (Matiang’I et al., 2016) as well absence of organizational justice, disrespectful behaviors, and conflict between personal values and values of the school were other reasons (Rostami et al., 2015).

Allahkerim (2020) expressed the key strategies to mitigate burnout. According to Allahkerim (2020) teachers should take intensive efforts to discover the individual and academic challenges they might encounter, focus their hobbies in their free time, request a clear and understandable job description from the administrators and ask for in-service training to keep themselves updated regarding the advancements at teaching profession.

Numerous studies, both nationally and internationally (Acar and Çoğaltay, 2021; Adıgüzel, 2016; Allahkerim, 2020; Demirel and Cephe, 2015; Hatoka et al., 2023; Jamaludin and You, 2019; Özgül and Polat, 2018; Özgül and Atan, 2016; Sadeghi and Kezrlou, 2016; Shaheen and Mahmood, 2016), have aimed to explore educators’ level of burnout based on socio-demographic variables. Furthermore, several studies have explored teachers’ attitudes towards organizational cynicism (Abdurrezzak et al., 2023; Aksu et al., 2024; Gedik and Üstüner, 2019; Kalağan and Güzeller, 2010; Vatansever-Bayraktar and Mutlu, 2023) via demographic variables. However, limited correlational studies have examined the relationship between teachers’ burnout and organizational cynicism (Akan and Yarım, 2019; Amasralı and Aslan, 2017; Duman et al., 2020).

In light of all these points, the hypotheses of this study are as follows:

1. Do participants significantly differ in their levels of organizational cynicism and burnout according to gender?
2. Do participants significantly differ in their levels of organizational cynicism and burnout according to age?
3. Do participants significantly differ in their levels of organizational cynicism and burnout according to marital status?
4. Do participants significantly differ in their levels of organizational cynicism and burnout according to educational status?
5. Do participants significantly differ in their levels of organizational cynicism and burnout according to years of professional experience?
6. Is there a significant relationship between burnout and organizational cynicism?
7. Does burnout predict organizational cynicism?

## 2 Materials and methods

Data were expressed as numerical values, and statistical analysis was conducted to interpret the findings. A quantitative research design was employed to examine the research questions (Karasar, 2016).

This study aimed to examine the correlations between burnout and organizational cynicism among teachers who worked in public secondary schools. Thus, this study employed a correlational descriptive research model that investigated the correlation between two or more variables (Büyüköztürk, 2018).

### 2.1 Sample

A total of 3,018 secondary school teachers actively taught during the 2023–2024 spring semester in Turkish Republic of Northern Cyprus (TRNC) public schools (KKTC Eğitim Ortak Hizmetler Dairesi Müdürlüğü, 2022). This study planned to recruit 345 secondary school teachers, obtained from a predicted sample Table (95% confidence interval, %5 standard error) introduced by Çıngı in 1994 (Çıngı, 1994). Moreover, a convenient sampling method was used to select the participants. Researchers choose those who were easily accessible as well as convenient (Gravetter and Forzano, 2012).

Participant consent was obtained online via Google Forms, and all participants voluntarily took part in the study. No groups were adversely affected, and confidentiality was strictly maintained.

### 2.2 Measures

Data collection comprised three sections: personal information form, the Maslach Burnout Inventory, and Organizational Cynicism Scale. Also Cronbach’s alpha coefficient was calculated to determine the internal consistency of the scales. The values obtained from the analyses indicate that the scales have a high level of reliability. For instance, the Cronbach’s alpha value was found to be (*α* = 0.74) for the organizational cynicism scale and (α = 0.76) for the burnout scale. These findings support that the measurement instruments used in the study are valid and reliable.

#### 2.2.1 Personal information form

The form was designed to obtain teachers’ socio-demographic profile and comprised five questions regarding their gender, age, marital status, academic background, and seniority.

##### 2.2.1.1 Maslach burnout inventory (MBI)

Maslach and Jackson (1981) developed the Maslach Burnout Inventory (MBI) that comprised 22 items on three sub-dimensions: emotional exhaustion, depersonalization and reduced personal accomplishment (eight items). Responses were rated on a 5-point Likert Scale that ranged from 0 = “Never,” 1 = “Rarely,” 2 = “Sometimes,” 3 = “Often,” 4 = “Very Often.” Table 1 presents the details of the sub-dimensions and items.

**Table 1:** Details of burn out scale.

Dimensions of the scale	Items
Emotional exhaustion	1,2,3*,6*,8,13,16,20*
Depersonalization	5,10,11,15,21,22
Reduced personal accomplishment	4,7,9,12,14,17,18,**19**

* Items 3, 6, 19, 20 will be reversed.

##### 2.2.1.2 Organizational cynicism scale

The Organizational Cynicism Scale, developed by Brandes et al. (1998), comprised 13 items on three sub-dimensions. Responses were rated on a 5-point Likert scale (1 = Strongly Disagree, 5 = Strongly Agree). Table 2 presents the details regarding the items and sub-dimensions.

**Table 2:** Details of organizational cynicism scale.

Dimensions of the scale	Items
Cognitive	1–5
Affective	6–9
Behavioral	10–13

## 3 Results

Table 3 presents the participants’ socio-demographic profile. The first five items of the questionnaire asked for “Socio-demographic Data,” which included gender, age, academic background, marital status, and seniority, respectively. Of the respondents, 72.7% were female and 27.8% were male, as shown in Table 1. Regarding age distribution, most respondents (50.3%) were at least 41 years old, while 19.10% were aged 21–30 years. Furthermore, 35.6 and 64.4% had a Master’s and Bachelor’s degree, respectively. In addition, 65.3% were married and 34.7% were single. Of the respondents, 18.6, 17.2, 16.1, and 48.1% had 1–5, 6–10, 11–15, and a minimum 16 years of experience in teaching profession, respectively.

**Table 3:** Socio-demographic profile of the respondents.

Gender	*N*	%
Female	399	72.2
Male	154	27.8
Age		
21–30	106	19.1
31–40	169	30.6
41 and above	278	50.3
Education Level		
Bachelor’s	356	64.4
Master’s	197	35.6
Marital		
Married	361	65.3
Single	192	34.7
Seniority		
1–5 years	103	18.6
6–10 years	95	17.2
11–15 years	89	16.1
16 years and above	266	48.1
Total	**553**	**100**

Bold values indicates a statistically significant difference at *p* < 0.05. *N* = frequency. % = percentage.

Table 4 summarizes the mean burnout scores. Participants experienced intense burnout in the dimension of reduced personal accomplishment (X̅=22.53, SD = 2.78).

**Table 4:** Mean score results-burn out dimensions.

Burn-out dimensions	X̅	SD
Depersonalization	6.77	2.65
Emotional exhaustion	15.62	2.45
Reduced personal accomplishment	22.33	2.78

Table 5 summarizes the mean organizational cynicism scores results. Results revealed that cognitive dimension had the highest mean score (X̅=14.21, SD = 1.71).

**Table 5:** Mean score results-organizational cynicism.

Organizational cynicism dimensions	X̅	SD
Behavioral	7.04	1.88
Affection	11.01	1.64
Cognitive	14.21	1.71

Table 6 presents the normality test results of two the scales. Field (2009) indicated that *p*_sig_ < 0.05 indicated that non-parametric statistical tests should be employed to assess the hypothesis. However, skewness and kurtosis values also played a vital role in examining whether the data had normal distribution. Cevahir (2020) stated that that if the values of the skewness was + and >1, the data were skewed to the right. Furthermore, positive kurtosis values indicated sharpness of the data. Therefore, participants’ data were not normally distributed; instead, data were skewed to the right side and had sharpness. Thus, non-parametric statistical tests were employed.

**Table 6:** Normality test results.

Dimensions	Kolmogorov–Smirnov				
	Statistic	df	Sig.	Skewness	Kurtosis
Cognitive	0.065	552	0.000	1.207	2.225
Affective	0.117	552	0.000	1.321	1.941
Behavioral	0.113	552	0.000	1.265	1.205
Emotional exhaustion	0.121	552	0.000	1.377	3.310
Depersonalization	0.171	552	0.000	1.461	2.268
Reduced personal accomplishment	0.165	552	0.000	1.353	1.936

Teachers had statistically significant depersonalization and reduced personal accomplishment dimensions (*p* < 0.05). Precisely, burnout levels in male teachers were higher compared with their female colleagues in the depersonalization dimension.

Table 7 presents the results of the Mann–Whitney *U* test conducted to explore the statistical significance of teachers’ organizational cynicism score according to their gender. Results demonstrated statistically significant differences in affective and behavioral dimensions (*p* < 0.05). Hence, male teachers had high organizational cynicism levels in the affective and behavioral cynicism dimensions compared with their female colleagues.

**Table 7:** Mann–Whitney *U*-test results on burnout score differences of participants’ gender.

Dimensions	Gender	*N*	Mean rank	Sum of ranks	*U*	*p*
Emotional exhaustion	Female	399	279,28	111,434,50	29,811,500	0.585
	Male	154	271,08	41,746,50		
Depersonalization	Female	399	264,00	105,337,50	25,537,500	0.002*
	Male	154	310,67	47,843,50		
Reduced personal accomplishment	Female	399	286,08	114,147,50	27,098,500	0.751*
	Male	154	253,46	39,033,50		

The asterisk (*) in the tables indicates that a significant difference was found as a result of the test.Bold values indicates a statistically significant difference at *p* < 0.05. *N* = frequency. % = percentage.

Kruskal-Wallis *H* test results revealed that teachers were statistically significant differences in reduced personal accomplishment dimension according to their age. To determine statistical significance between age groups, Tamhane’s T2 test was employed. Results revealed that teachers aged 21–30 years had statistically significant differences compared with those aged 31–40 and >41 years. Teachers aged 21–30 years had the lowest burnout level in the reduced personal accomplishment dimension compared with those aged 31–40 and >41 years.

Table 8 presents the Mann–Whitney U test results comparing organizational cynicism scores by gender. The findings indicate no significant difference in the cognitive dimension, while significant differences were observed in the affective and behavioral dimensions, suggesting gender-based variation in certain aspects of organizational cynicism among TRNC secondary public school teachers.

**Table 8:** Mann–Whitney *U*-test results on organizational cynicism score differences of participants’ gender.

Organizational cynicism	Gender	*N*	Mean rank	Sum of ranks	*U*	*p*
Cognitive	Female	399	275,91	110,089,50		
	Male	154	279,81	43,091,50	30,289,500	0.797
Affective behavioral	Female	399	268,10	106,972,00		
	Male	154	300,06	46,209,00	27,172,000	0.035*
	Female	399	266,59	106,367,50		
	Male	154	303,98	46,813,50	26,567,500	0.010*

The asterisk (*) in the tables indicates that a significant difference was found as a result of the test.Bold values indicates a statistically significant difference at *p* < 0.05. *N* = frequency. % = percentage.

Participating teachers had no statistically significant differences (*p* > 0.05) regarding their age at context of organizational cynicism, as shown in Table 9.

**Table 9:** Kruskal–Wallis *H*-test results on burnout score differences of participants’ age.

Dimensions	Age	N	Mean rank	df	*χ* ^2^	*p*
Emotional exhaustion	21–30 (1)	106	297,63	2	2,810	0,245
	31–40 (2)	169	279,50			
	41 and above (3)	278	267,62			
Depersonalization	21–30 (1)	106	283,17	2	2,232	0,891
	31–40 (2)	169	273,75			
	41 and above (3)	278	276,62			
Reduced personal accomplishment	21–30 (1)	106	232,99	2	10,076	0.000*
	31–40 (2)	169	285,20			1–2,1–3
	41 and above	278	288,80			

The asterisk (*) in the tables indicates that a significant difference was found as a result of the test.Bold values indicates a statistically significant difference at *p* < 0.05. *N* = frequency. % = percentage.

Table 10 presents the Kruskal–Wallis H test results comparing organizational cynicism scores by age. The results show no statistically significant differences across age groups in any of the dimensions, indicating that organizational cynicism levels are similar among teachers of different ages.

**Table 10:** Kruskal–Wallis *H*-test results on organizational cynicism score differences of participants’ age.

Dimensions	Age	*N*	Mean rank	df	*χ* ^2^	*p*
Cognitive	21–30 (1)	106	275,54			
	31–40 (2)	169	263,88	2	1,949	0,377
	41 and above (3)	278	285,53			
	21–30 (1)	106	283,04	2	2,526	0,283
Affective	31–40 (2)	169	260,78			
	41and above	278	284,56			
	21–30 (1)	106	273,64	2	0,713	0,700
Behavioral	31–40 (2)	169	270,27			
	41and above	278	282,38			

Table 11 presents the results of Mann–Whitney *U* test conducted to assess whether the participating teachers had statistically significant differences regarding their education level in the context of burnout levels. Teachers with master’s degree had higher burnout levels in all the dimensions compared with their colleagues with Bachelor’s degree.

**Table 11:** Mann–Whitney *U*-test results on burnout score differences of participants’ education level.

Dimensions	Education level	*N*	Mean rank	Sum of ranks	*U*	*p*
Emotional exhaustion	Bachelor’s	356	273,12	97,232,00	33,686,000	0.000*
	Master’s	197	284,01	55,949,00		
Depersonalization	Bachelor’s	356	273,65	97,418,50	33,872,500	0.002*
	Master’s	197	283,06	55,762,50		
Reduced personal accomplishment	Bachelor’s	356	257,64	91,721,50	28,175,500	0.000*
	Master’s	197	311,98	61,459,50		

The asterisk (*) in the tables indicates that a significant difference was found as a result of the test.Bold values indicates a statistically significant difference at *p* < 0.05. *N* = frequency. % = percentage.

Table 12 presents the results of the Mann–Whitney *U* test conducted to assess whether the teachers had statistically (*p* < 0.05) significant differences regarding their education level in context of organizational cynicism levels. Teachers with Master’s degree had higher organizational cynicism levels in all the dimensions compared with teachers with Bachelor’s degree.

**Table 12:** Mann–Whitney *U*-test results on organizational cynicism score differences of participants’ education level.

Dimensions	Education level	*N*	SO	ST	*U*	*p*
Cognitive	Bachelor’s	356	265,19	100,939,50		
	Master’s	197	283,54	52,241,50	26,491,000	0.011*
Affective behavioral	Bachelor’s	356	273,16	99,369,00		
	Master’s	197	279,13	53,812,00	25,958,500	0.000*
	Bachelor’s	356	275,66	98,134,50		
	Master’s	197	279,42	55,046,50	25,600,000	0,023

The asterisk (*) in the tables indicates that a significant difference was found as a result of the test.Bold values indicates a statistically significant difference at *p* < 0.05. *N* = frequency. % = percentage.

Based on the Mann–Whitney test results, participating teachers had statistically significant differences in (*p* < 0.05) the reduced personal accomplishment dimension. Results revealed that married teachers had higher burnout levels compared with single teachers.

Findings revealed no statistical significance differences (*p* > 0.05) between teachers regarding their marital status in context of organizational cynicism.

Kruskal-Wallis *H* test results revealed that participating teachers had statistically significant differences in the emotional exhaustion dimension regarding seniority. Therefore, Tamhane’s post-hoc test was conducted to examine the main variables regarding differences. Teachers with 1–5 years of work experience had statistically significant differences compared with teachers with a minimum of 16 years. Teachers with 1–5 years of work experience tended to experience intensive burnout in the emotional exhaustion dimension compared with those with a minimum of 16 years.

Table 13 presents the Mann–Whitney U test results examining differences in burnout scores by marital status. The analysis indicates that emotional exhaustion and depersonalization did not differ significantly between married and single teachers. However, a statistically significant difference was observed in reduced personal accomplishment, with married teachers exhibiting higher levels, suggesting marital status may influence this dimension of burnout.

**Table 13:** Mann–Whitney *U*-test results on burnout score differences of participants’ marital status.

Dimensions	Marital status	*N*	Mean rank	ST	*U*	*p*
Emotional exhaustion	Married	361	273,54	98,746,50	33,405,500	0.481
	Single	192	283,51	54,434,50		
Depersonalization	Married	361	276,72	99,894,50	34,553,500	0.954
	Single	192	277,53	53,286,50		
Reduced personal accomplishment	Married	361	291,33	105,169,00	29,484,000	0.004*
	Single	192	250,06	48,012,00		

The asterisk (*) in the tables indicates that a significant difference was found as a result of the test.Bold values indicates a statistically significant difference at *p* < 0.05. *N* = frequency. % = percentage.

Table 14 presents the Mann–Whitney U test results assessing differences in organizational cynicism scores according to marital status. The analysis revealed no statistically significant differences between married and single teachers across the cognitive, affective, or behavioral dimensions, indicating that marital status does not appear to exert a significant influence on organizational cynicism among the participants.

**Table 14:** Mann–Whitney *U*-test results on organizational cynicism score differences of participants’ marital status.

Dimensions	Marital status	*N*	SO	ST	*U*	*p*
Cognitive	Married	361	279,01	100,722,50		
	Single	192	273,22	52,458,500	33,930,500	0.684
Affective behavioral	Married	361	270,04	97,484,00		
	Single	192	290,09	55,697,00	32,143,000	0.159
	Married	361	276,17	99,697,50		
	Single	192	278,56	53,483,50	34,356,500	0.862

Table 15 presents the results of the Kruskal–Wallis *H* test conducted to assess whether the participating teachers had statistically significant differences regarding their seniority in context of organizational cynicism level. Teachers had statistically significant differences in the cognitive and affective dimensions (*p* < 0.05). To assess the main variables of differences between seniority of teachers, Tamhane’s post-hoc test was conducted. Teachers with 6–10 years of work experience had statistically significant differences in both dimensions compared with those with 11–15 years of experience. Hence, teachers with 6–10 years tended to exhibit intense cynic behaviors in both dimensions compared with their colleagues who with 11–15 years of experience.

**Table 15:** Kruskal–Wallis *H*-test results on burnout score differences of participants’ seniority.

Dimension	Seniority	*N*	Mean rank	df	*χ* ^2^	*p*
Emotional exhaustion	1–5 years (1)	103	288,24	3	6,800	0.000*
	6–10 years (2)	95	265,19			1–4
	11–15 years (3)	89	311,80			
	16 years and above (4)	266	265,22			
Depersonalization	1–5 years (1)	103	258,68	3	5,855	0.119
	6–10 years (2)	95	309,42			
	11–15 years (3)	89	282,53			
	16 years and above (4)	266	270,66			
Reduced personal accomplishment	1–5 years (1)	103	222,45	3	17,767	0.121
	6–10 years (2)	95	277,04			
	11–15 years (3)	89	271,32			
	16 years and above (4)	266	300,01			

The asterisk (*) in the tables indicates that a significant difference was found as a result of the test.Bold values indicates a statistically significant difference at *p* < 0.05. *N* = frequency. % = percentage.

Table 16 presents the Kruskal–Wallis H test results examining differences in organizational cynicism scores based on participants’ seniority. The analysis indicates significant differences in the cognitive and affective dimensions, particularly between teachers with 6–10 years and 11–15 years of experience, while no significant difference was observed in the behavioral dimension. These findings suggest that seniority may influence certain aspects of organizational cynicism among teachers.

**Table 16:** Kruskal–Wallis *H*-test results on organizational cynicism score differences of participants’ seniority.

Dimensions	Seniority	*N*	Mean rank	SD	*χ* ^2^	*p*
Cognitive	1–5 years (1)	103	266,12			
	6–10 years (2)	95	250,12	3	5,619	**0.002**
	11–15 years (3)	89	273,11			(2–3)
	16 years and above (4)	266	292,11			
	1–5 years (1)	103	266,37	3	3,055	0.015
Affective	6–10 years (2)	95	269,47			(2–3)
	11–15 years (3)	89	261,30			
	16 years and above (4)	266	289,06			
	1–5 years (1)	103	262,66	3	1,977	**0.577**
Behavioral	6–10 years (2)	95	276,08			
	11–15 years (3)	89	293,95			
	16 years and above (4)	266	277,21			

Bold values indicates a statistically significant difference at *p* < 0.05. *N* = frequency. % = percentage.

Table 17 presents the Spearman rank correlation results between organizational cynicism and burnout. The analysis indicates a positive and statistically significant correlation (ρ = 0.315, p < 0.01), suggesting that higher levels of organizational cynicism are associated with higher levels of burnout among the participants.

**Table 17:** Spearman rank correlation result.

Organizational cynicism-burn out correlation result	
Organizational cynicism	1
Two tailed correlation (*p*)	0.000**
Burn out	**0.315**

The asterisk (*) in the tables indicates that a significant difference was found as a result of the test.Bold values indicates a statistically significant difference at *p* < 0.05. *N* = frequency. % = percentage.

Table 18 presents the results of Spearman’s rank correlation. A significant, positive and weak two-tailed correlation was observed between organizational cynicism and burnout. Hence, these two notions were inter-related and acted as a product of each other.

**Table 18:** Simple linear regression test result.

Dependent variable: burn out			
Independent variable	Beta (*β*)	*T*-value	*p*-value
Organizational cynicism	1,195	7,431	0.000*

	*R*	*R* ^2^	*F*-value
Results of R, *R*^2^ ve *F* values	0.315	0.099	34,711

The asterisk (*) in the tables indicates that a significant difference was found as a result of the test.Bold values indicates a statistically significant difference at *p* < 0.05. *N* = frequency. % = percentage.

Simple linear regression analysis revealed that organizational cynicism had a positive impact on burnout. It predicted the total variance of burnout by 9.9%.

## 4 Discussion

Several studies have explored the significance of burnout levels on teachers via socio-demographic variables. Şanlı and Tan (2017) explored its statistical significance on the dimension of reduced personal dimension, which was consistent with the findings of this study. A main reason of this finding could be that males tended to have a less fragile nature and were more likely to exhibit aggressive behaviors compared with females. Thus, they tended to face intensive burnout in the depersonalization dimension. Burnout literature revealed that studies that assessed the statistical significance of age reported conflicting results. Some found no statistical significance (Acar and Çoğaltay, 2021; Özgül and Altan, 2016; Shaheen and Mahmood, 2016), while other (Birkan, 2020; Cabuk, 2015; Özgül and Polat, 2018) found a statistical significance. Findings of this study were compatible with those of previous research. Teachers aged 21–30 years have recently graduated from teacher training schools and have additional knowledge of modern education and training techniques, effective use of educational resources consistent with the age, content of modern pedagogical formation, and contemporary classroom management, compared with their older colleagues. Thus, they tend to have lower burnout levels in the reduced personal accomplishment dimension. Education level is a prominent socio-demographic variable that impacted burnout. Most researchers (Allahkerim, 2020; Sadeghi and Khezrlou, 2016) observed positive relationships between education level and level of burnout. In this study, results revealed that teachers with Master’s degree tended to experience intensive burnout in all the dimensions. This could be since teachers with Master’s degree were appointed to administrative positions earlier than their colleagues with Bachelor’s degree. However, teachers with Master’s degree may question themselves regarding their professional knowledge, abilities, and experiences; Furthermore, they may believe that they will not be successful while performing these administrative roles and/or responsibilities, which can result in higher burnout levels in reduced personal accomplishment. Various studies aimed to determine the association between marital status and burnout levels. Most observed that married individuals were more likely to encounter minimum burnout as couples can build communication between each other regarding their work-related problems and recommend or perform activities together, such as yoga or mediation techniques, which can lower their burnout levels. However, in this study, married teachers experienced higher burnout levels in the reduced personal accomplishment dimension. This could be due to their concern of losing their professional knowledge, abilities and self-confidence owing to chronic work stress as well as worries of not being able to advance in their careers. Furthermore, not being able to contribute to the family budget may be a factor. The emergence of such finding can be due to the fact that teachers who have just started to teaching profession may feel insufficiently experienced in mitigating challenges (i.e., students with discipline problems, attitudes of the senior colleagues towards to them, professional knowledge to discover readiness level of the learners, difficulties in shaping exam questions consistent with the curriculum). Furthermore, they may not have sufficient work experience and all these challenges may lead them to experience intense burnout in the emotional exhaustion dimension.

Numerous studies have explored the association between gender and organizational cynicism. However, the results are contradictory. Some studies reported no statistical significance between gender and organizational cynicism (Aksu et al., 2024; Nartgün and Kalay, 2014) while others reported a statistical significance (Abdurrezzak et al., 2023; Vatansever-Bayraktar and Mutlu, 2023 and Mert, 2022). These studies concluded that females had high organizational cynicism levels. However, this study found that males tended to have higher organizational cynicism levels in the affective and behavioral cynicism dimensions. A main reason could be that male teachers tended to evaluate their friends and school leaders/administrators more negatively than their female colleagues. Thus, they were more like to criticize their school leaders/administrators and isolate themselves from their educational institution. Apart from gender, age was also a main socio-demographic factor. Many studies reported a positive relationship between age and organizational cynicism (Güneş, 2023; Mirvis and Kanter, 1991). However, this study observed no statistical significance between age and organizational cynicism, which indicated that participating teachers from different age groups had similar organizational cynicism levels.

Limited studies have examined statistical significances between marital status and level of organizational cynicism. Results were contradictory. Abdurrezzak et al. (2023) concluded no statistical significance between marital status and organizational cynicism level, whereas Vatansever-Bayraktar and Mutlu (2023) indicated that participating teachers were statistically significance regarding their marital status in context of organizational cynicism level. The results of this study was consistent with those of Abdurrezzak et al. (2023), as single and married teachers shared similar perception towards to organizational cynicism. Furthermore, this study found a positive relationship between level of education and organizational cynicism level, which was congruent with those of Aksu et al. (2024) and Ceylan (2022). The main reason was that teachers with Master’s degree might have higher expectations regarding career and advancement opportunities, salary, management style of school leaders/administrators, allowance of participating to decision-making process, and capabilities of their school leaders/administrators. A gap between their expectation and reality may result in teachers becoming upset and aggressive and more likely to exhibit cynic attitudes, reactions, and behaviors.

Similar to education level, seniority was a crucial factor that influenced the level of organization cynicism. O’Connell et al. (1986) argued an inverse relationship between seniority and organizational cynicism. Various scholars have attempted to assess relationship between seniority and level of organizational cynicism; however, they reported contradictory outcomes. Aksu et al. (2024) found no statistical significance between seniority and organizational cynicism. However, Culha and Kaya (2021) found a positive relationship between organizational cynicism and seniority. In this study, teachers with 6–10 years teaching experience had intensive cognitive and affective organizational cynicism compared with their colleagues with 11–15 years of experience. Hence, teachers with 11–15 years tended to have more experience of the nature and dynamics of teaching profession and knowledge of the educational stakeholders of their education institution. Therefore, they were more aware of shaping reasonable expectations from administrators and colleagues as well as educational expectations. All these factors play critical roles in diminishing their cynic behaviors in the related dimensions. Spearman’s rank correlation results demonstrated two-tailed, significant, positive correlation between two notions, which implicitly indicated that these two notions acted as a complement (product) with each other. Thus, the existence of organizational cynicism could trigger burnout, or generation of burnout could fuel organizational cynicism in educational institutions. However, limited studies have examined the correlation between these two notions. None investigated the two-tailed correlation between burnout and organizational cynicism. However, the correlation result of this study overlapped with those of previous researchers (Akan and Yarım, 2019; Amasralı and Aslan, 2017; Duman et al., 2020).

Based on these findings, school administrators to focus on in-service-training programs and direct teachers to elevate their professional knowledge. Hence, teachers may easily mitigate burnout, which may arise owing to reduced personal accomplishment. Furthermore, school administrators should build a sincere, welcoming organizational climate and organizational justice as well as should take intensive efforts to maximize organizational trust to lower cynicism at the cognitive dimension.

Future researchers should include teachers at different levels of education (i.e., primary and higher education levels) to determine the differences and similarities regarding burnout and organizational cynicism levels and socio-demographic variables. This study was limited to investigating the relationship between burnout and organizational cynicism. Future studies should extend the scope by adding other notions, such as intention to leave, organizational alienation, teachers’ self-efficacy, and management style of the school administrator, and measure their mediating roles on the correlation between organizational cynicism and burnout. Furthermore, they should employ mixed research study designs and conduct semi-structured interviews to obtain findings from different perspectives.

## 5 Conclusion and recommendations

This study investigated the relationship between organizational cynicism and teacher burnout among secondary public school teachers in the Turkish Republic of Northern Cyprus (TRNC), as well as the potential differences in these constructs across socio-demographic variables such as gender, age, marital status, education level, and professional experience. The findings revealed that burnout levels were influenced by all examined socio-demographic factors, whereas organizational cynicism showed significant variation primarily with gender, education level, and years of professional experience.

A positive and significant correlation between burnout and organizational cynicism was observed, and organizational cynicism explained 9.9% of the variance in burnout. Although this effect size is modest, it indicates that teachers’ negative attitudes toward administrators, colleagues, and students can exacerbate feelings of exhaustion and reduced personal accomplishment. These findings align with previous international studies suggesting that organizational cynicism contributes to lower teacher motivation, decreased engagement, and impaired overall school performance.

From a practical standpoint, the study highlights the importance of addressing organizational cynicism in schools. Interventions targeting the improvement of school climate, fostering positive teacher-administrator relationships, and promoting professional development may help reduce burnout. Additionally, attention to socio-demographic differences suggests that tailored support strategies may be particularly beneficial for specific teacher groups, such as those with higher seniority or certain educational backgrounds.

Despite the contributions of this study, limitations must be acknowledged. The study’s sample is limited to TRNC secondary public school teachers, which restricts generalizability. Future research with larger and more diverse samples, as well as longitudinal designs, is recommended to validate and expand upon these findings. Moreover, incorporating qualitative methods could provide deeper insights into the mechanisms linking organizational cynicism and burnout in educational settings.

In summary, this study provides valuable evidence on the interplay between organizational cynicism and burnout in a unique educational context, highlighting both theoretical implications for understanding teacher attitudes and practical strategies for improving teacher well-being and school performance.

*Also about recommendations*

Based on the findings of this study, it is recommended that school administrators implement programs aimed at reducing organizational cynicism and supporting teacher well-being. Professional development initiatives should focus on stress management, enhancing communication skills, and fostering positive teacher-administrator relationships. Tailored interventions may be particularly beneficial for specific teacher groups, considering differences in gender, education level, or professional experience. For future research, larger and more diverse samples are suggested, along with longitudinal or mixed-methods designs to better understand causal relationships. Additionally, qualitative studies could provide deeper insights into how organizational cynicism develops and affects burnout in educational settings.

Considering the study’s findings and its limitations, it is recommended that school administrators implement programs to reduce organizational cynicism and support teacher well-being. Professional development initiatives should focus on stress management, communication skills, and fostering positive teacher-administrator relationships. Tailored interventions may be particularly beneficial for specific teacher groups based on gender, education level, or professional experience. Given that the study was limited to TRNC secondary public school teachers and employed a cross-sectional design, future research should include larger and more diverse samples, longitudinal or mixed-methods approaches, and additional contextual factors, such as school climate and coping strategies, to better understand the dynamics between organizational cynicism and burnout.

## Data Availability

The raw data supporting the conclusions of this article will be made available by the authors, without undue reservation.
